# Associations of inflammation/nutrition-related indicators (RAR and MAR) with chronic kidney disease: evidence from NHANES 2005–2018

**DOI:** 10.3389/fnut.2026.1725996

**Published:** 2026-04-27

**Authors:** Huaiman Li, Ying He, Binhuan Chen, Zhao Liu, Zhouchun Zhu, Weiping Zhu, Shuangqin Chen

**Affiliations:** 1 Division of Nephrology, Department of Medicine, The Fifth Affiliated Hospital of Sun Yat-Sen University, Zhuhai, China; 2 Sanya Hospital of Traditional Chinese Medicine, Sanya, China; 3 Medical Equipment Department, The Fifth Affiliated Hospital of Sun Yat-Sen University, Zhuhai, China

**Keywords:** albumin, chronic kidney disease, inflammation/nutrition-based indicators, NHANES, population-based study

## Abstract

**Objective:**

Ratios of red blood cell distribution width over albumin (RAR) and monocyte count over albumin (MAR) have recently emerged as inflammation- and nutrition-related biomarkers, demonstrating promise has been widely examined for its connection with the manifestation and clinical impact of several diseases. The research seeks to explore a potential link between RAR and MAR and the prevalence of chronic kidney disease (CKD).

**Methods:**

A cross-sectional analysis was conducted on 27,072 participants from the 2005–2018 NHANES dataset, representative of the U. S. population. Analyses included univariate and multivariate logistic regression, restricted cubic spline modeling, stratified analyses with interaction testing, mediation analyses, and assessment of clinical utility using calibration plots and decision curve analyses.

**Results:**

The proportion of participants with CKD was found to be 17.7%. After adjusting for a broad set of covariates, logistic regression models with multiple covariates indicated that both RAR and MAR showed significant positive correlations with CKD, with individuals in the highest RAR category exhibiting the strongest associations (OR = 1.68, 95% CI: 1.56–1.80) and highest MAR group (OR = 1.33, 95% CI: 1.24–1.43) had increased odds of CKD. RCS modeling revealed notable nonlinear relationships between RAR and MAR with CKD (nonlinearity test, *p* < 0.001). Both biomarkers matched observed risks and offered positive net clinical benefit. A significant modification by sex of the link of RAR and CKD prevalence was also detected (interaction *p* < 0.001). Mediation analyses further indicated that hemoglobin and neutrophil-to-lymphocyte ratio (NLR) partially mediated the associations of RAR and MAR with CKD, respectively. Sensitivity analyses produced consistent outcomes, supporting the robustness of these findings. These findings were further corroborated in an independent hospital-based validation cohort, which showed consistent directions of association of RAR and MAR with CKD.

**Conclusion:**

Higher levels of the inflammation- and nutrition-related markers RAR and MAR show a link to an increased prevalence of CKD, with these associations partially mediated by hemoglobin and NLR and moderated by sex. Given their simplicity and low cost, RAR and MAR could serve as potential markers for detecting CKD among individuals, although the cross-sectional design precludes causal inference or predictive conclusions.

## 1 Introduction

Chronic kidney disease (CKD) has become a rising worldwide health burden (1). Approximately 84.36 million people were estimated to have CKD in 2017 (2, 3), and this number continues to rise. Estimates derived from the Global Burden of Disease (GBD) study indicate 18,986,903 new CKD cases were reported in 2019 (4). In 2021, CKD accounted for a significant portion of global mortality, placing it 11th among age-standardized causes of death (5).

Evidence from multiple studies suggests that inflammatory processes significantly influence both the initiation and advancement of CKD (6, 7), as monocytes significantly contribute to the inflammatory response (8, 9). Poor nutritional status has been associated with CKD development (10, 11), and reduced serum albumin levels are associated with a higher likelihood of CKD development (12). Serum albumin by itself may not accurately indicate nutritional status in CKD, because as a negative acute-phase protein, it undergoes a reduction during systemic inflammatory responses regardless of actual protein–energy depletion. Likewise, biomarkers reflecting markers of inflammation, such as the neutrophil-to-lymphocyte ratio (NLR) primarily reflect immune activation without accounting for concurrent nutritional alterations. Recent studies suggest that composite biomarkers integrating measures of inflammation and nutritional status provide a comprehensive assessment in chronic diseases (13). A nutritional inflammation index, integrating albumin and leukocyte-derived measures, was closely associated with mortality in sepsis patients and showed stronger correlations than its individual elements in multivariable analyses (14), providing support for the use of integrated inflammation–nutrition ratios.

Red cell distribution width (RDW) reflects systemic inflammation indirectly (15, 16), additionally provides information on nutritional status and anemia (17). RDW-to-albumin ratio (RAR) is increasingly considered a valuable predictor of clinical outcomes across several disorders, including Parkinson’s disease, cardiovascular diseases, and CKD (18–21). Beyond CKD, inflammation–nutrition composite ratios have also been examined in chronic metabolic and cardiovascular conditions. In a nationally representative U. S. population, RAR was significantly associated with metabolic syndrome and insulin resistance, with consistent dose–response patterns observed across exposure categories (22). Similarly, monocyte-based composite indices have been associated with renal outcomes; in a large population-based study, the monocyte-to-high-density lipoprotein ratio was independently related to CKD presence after multivariable adjustment (23). In people with diabetes or prediabetic conditions, the C-reactive protein–albumin–lymphocyte (CALLY) index, an integrated marker reflecting inflammation, nutritional, and immune function, showed an inverse correlation with both death from any cause and from cardiovascular disease in a cohort drawn from the general population (24). These findings collectively highlight the potential utility of composite inflammation–nutrition markers in diverse cardiometabolic conditions.

However, few studies have directly examined how composite measures reflecting status of inflammation and nutrition particularly RAR and the monocyte–to–albumin ratio (MAR) show a relationship with CKD. We utilized cross-sectional data from the U. S. National Health and Nutrition Examination Survey (NHANES 2005–2018) to explore this research question, and the main findings were later confirmed in an independent hospital cohort from the Nephrology Department at the Fifth Affiliated Hospital of Sun Yat-sen University. Our hypothesis was that increased RAR and MAR levels could relate to a greater prevalence of CKD among adults. Identifying robust and readily available inflammation–nutrition biomarkers may enhance clinical characterization of CKD status in population-based settings.

## 2 Materials and methods

### 2.1 Population sample

Data for this study were sourced from the NHANES dataset, covering seven successive publicly available cycles conducted between 2005 and 2018. NHANES constitutes conducted among the noninstitutionalized civilian population in the U. S. by the Centers for Disease Control and Prevention (CDC), designed to provide a comprehensive assessment of health and nutrition in the U. S. population through structured questionnaires, physical examinations, and laboratory evaluations. Approval from the NCHS Ethics Review Board was secured for this study, with all participants giving written informed consent. The NHANES official platform provides public access to all datasets employed in this analysis.^1^

**Figure 1:**
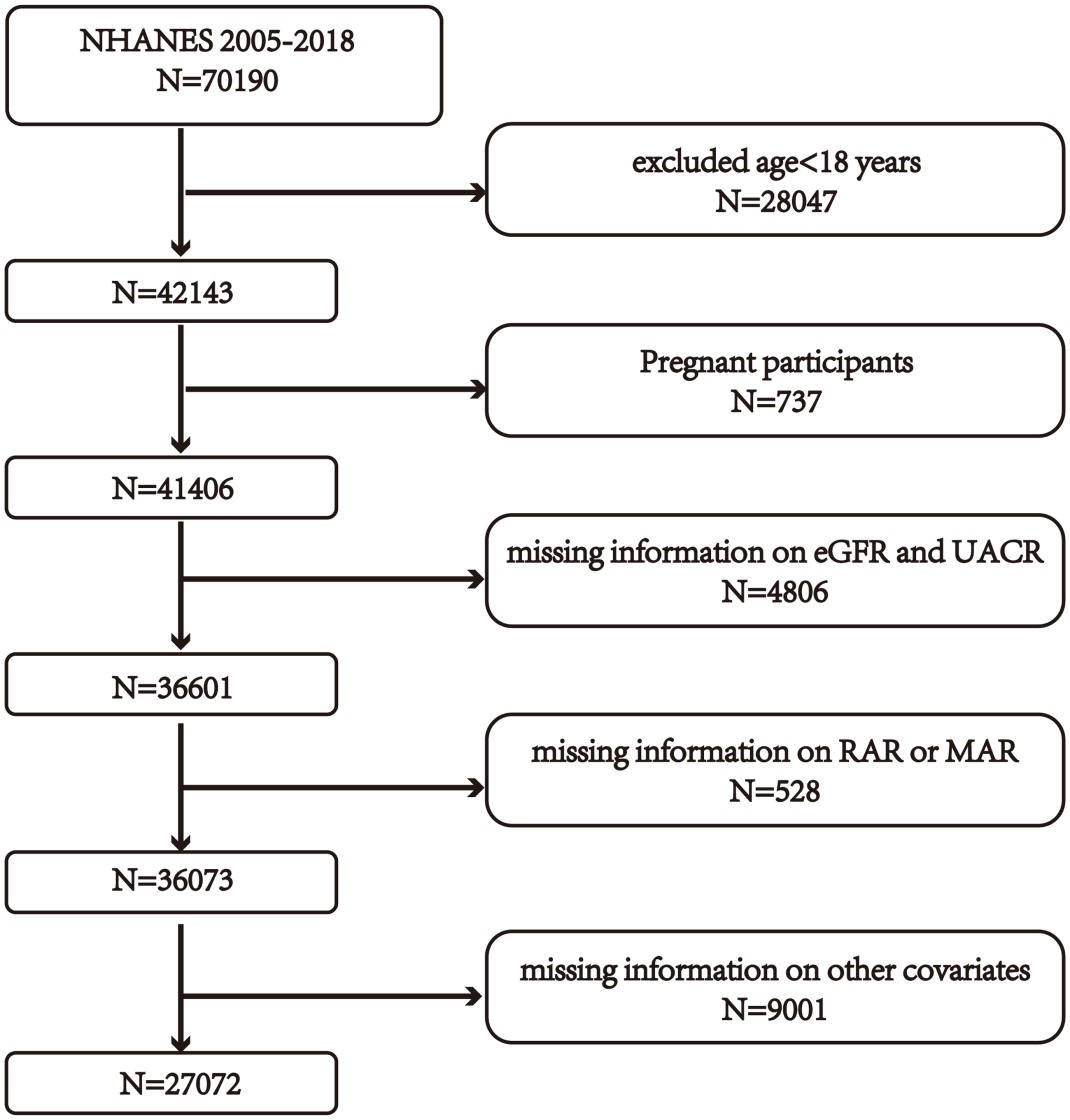
Flow diagram of participant selection from NHANES 2005–2018. Scr, serum creatinine; UACR, urinary albumin-to-creatinine ratio; CKD, chronic kidney disease; RAR, red cell distribution width-to-albumin ratio; MAR, mean corpuscular volume-to-red blood cell count ratio.

This study analyzed the associations among the indicators reflecting inflammatory and nutritional status, particularly red cell distribution width-to-albumin ratio (RAR) and the mean corpuscular volume to monocyte-to-albumin ratio (MAR)—with the presence of chronic kidney disease (CKD), we initially screened 70,190 individuals from the specified NHANES survey years. Participants were excluded applying the criteria outlined below: individuals younger than 18 years (*n* = 28,047); pregnant women (*n* = 737); those lacking key renal function indicators such as serum creatinine (Scr) or urinary albumin-to-creatinine ratio (UACR) (*n* = 4,806); individuals without available RAR or MAR values (*n* = 528); and cases missing essential covariate information, including demographic characteristics, lifestyle behaviors, and clinical measures (*n* = 9,001). After applying these criteria, 27,072 subjects met the eligibility requirements for the final statistical analysis. An overview regarding participant recruitment procedure is presented in Figure 1.

To independently validate the associations observed in the NHANES analysis, a hospital-based cohort was established using clinical data from Nephrology Department at the Fifth Affiliated Hospital of Sun Yat-sen University, Zhuhai, China. Patients admitted or visiting the nephrology department between January 2018 and December 2024 were identified through retrospective review. CKD cases were identified according to the KDIGO diagnostic criteria, while non-CKD patients—comprising individuals with conditions such as urinary tract infection, acute nephritis, or other non-chronic renal diseases—served as controls. Patients with incomplete laboratory or clinical data were excluded. After quality control, the validation analysis included 1,507 patients.

### 2.2 Exposure variables

The Coulter® DxH 800 automated hematology analyzer quantified white blood cell (WBC) counts, while neutrophil percentages were determined through the Coulter VCS system. RAR was calculated as the red cell distribution width-to-serum albumin ratio (g/dL); the MAR was derived from the monocyte count ratio (1000 cells/μL) to serum albumin concentration (g/dL). These two ratios were used to comprehensively assess systemic inflammation and nutritional status.

### 2.3 Outcome variable

CKD was diagnosed in accordance with the 2012 guidelines for medical practice from Kidney Disease: Improving Global Outcomes (KDIGO) (25), defined by an estimated glomerular filtration rate (eGFR) < 60 mL/min/1.73 m^2^ or clinical evidence of renal injury, such as albuminuria. The Chronic Kidney Disease Epidemiology Collaboration (CKD-EPI) equation was applied to participants’ serum creatinine (Scr) levels to derive eGFR. An eGFR < 60 mL/min/1.73 m^2^ was used to indicate reduced renal function (26), which corresponds to stage G3a or higher in the CKD classification. UACR, representing the ratio of albumin to creatinine in urine, was used to evaluate albuminuria, with levels ≥ 30 mg/g considered clinically significant (22). Urinary albumin levels were determined using a solid-phase fluorescent immunoassay, while urine creatinine concentrations were quantified by an enzymatic assay.

### 2.4 Covariates

Potential confounders were selected *a priori* based on established risk factors for CKD and findings from prior epidemiological studies. Multivariable adjustment models included demographic characteristics, lifestyle behaviors, and relevant clinical comorbidities. In the primary NHANES analyses, models included adjustment covariates considered in this study encompassed demographic, socioeconomic, and lifestyle factors such as age, sex, race/ethnicity, marital status, education, poverty-to-income ratio (PIR), body mass index (BMI), smoking, alcohol consumption, hypertension, diabetes, liver disease, and cancer. In the validation cohort associated with the Fifth Affiliated Hospital of Sun Yat-sen University, due to data limitations, only a subset of covariates was available for adjustment. Covariates considered encompassed demographic factors (age, sex), anthropometric measure (BMI), lifestyle behaviors (smoking status, alcohol consumption), and comorbidities including hypertension, diabetes, liver disease, and cancer.

Demographic variables (27) encompassed age and BMI. BMI was calculated as body weight divided by height squared (kg/m^2^) and categorized into four groups: underweight (<18.5 kg/m^2^), normal (18.5–23.9 kg/m^2^), overweight (24.0–27.9 kg/m^2^), and obese (≥28.0 kg/m^2^). Race/ethnicity was classified into five categories: non-Hispanic White, Mexican American, non-Hispanic Black, other Hispanic, or other/multiracial. Educational attainment was classified into three levels: less than high school, high school graduate, and beyond high school. Marital status was classified as married/cohabiting or unmarried, with the latter including widowed, divorced, or separated participants.

Lifestyle factors, including alcohol consumption, smoking, and physical activity, were classified into categories. Information on alcohol intake was obtained from two separate 24-h dietary recall interviews; individuals reporting alcohol use on either occasion were classified as alcohol users. Participants were classified as never smokers (<100 cigarettes in their lifetime), former smokers (≥100 cigarettes but not currently smoking), or current smokers (≥100 cigarettes and currently smoking). Physical activity was assessed using self-reported involvement in activities of vigorous intensity (e.g., running, basketball) or moderate-intensity (e.g., brisk walking, swimming, cycling), categorized into four levels: physically inactive, moderately active, vigorously active, and engaging in both moderate and vigorous activities. The poverty income ratio (PIR) was classified into three tiers: low (< 1.0), medium (1.0–3.0), and high (> 3.0).

Clinical comorbidities considered included hypertension, diabetes, liver disease, and cancer (28–30). The classification of hypertension relied on self-reported physician diagnosis together with measured blood pressure readings. Diabetes was defined as a self-reported prior diagnosis, HbA1c ≥ 6.5%, or fasting plasma glucose ≥ 126 mg/dL in laboratory tests. Liver disease and were ascertained using self-reported medical history (31).

The analysis incorporated several laboratory measures, including monocyte count, RDW, albumin, hemoglobin, RAR, MAR, eGFR, UACR, and the NLR.

### 2.5 Statistical analysis

Baseline characteristics of the study population, including demographic information, health behaviors, clinical indicators, and laboratory parameters, were summarized by relevant stratification factors. Continuous variables are expressed as means ± standard deviations (SDs), and categorical variables are presented as counts and percentages. For continuous variables, group differences were examined using weighted t-tests when comparing two groups and weighted linear regression for multiple-group comparisons, with overall significance assessed using Wald tests. Group differences in categorical variables were examined with weighted chi-square tests. All statistical analyses incorporated the complex survey design of NHANES, taking into account sampling weights, clustering, and stratification.

The ability of RAR and MAR, which reflect inflammation and nutritional status, to identify individuals with CKD was assessed using Receiver operating characteristic (ROC) curves. Using the Youden Index, optimal thresholds for RAR and MAR were established, and participants were grouped into high and low categories to assess CKD status. Associations between RAR, MAR, and CKD were examined using multivariable logistic regression, with CKD presence as the dependent variable. The analysis was conducted using three models: Model 1 unadjusted; Model 2 adjusted for demographic and lifestyle covariates (age, sex, race/ethnicity, education, PIR, smoking, alcohol use, physical activity, BMI); and Model 3 additionally controlled for clinical conditions including diabetes, hypertension, liver disorders, and cancer. Sensitivity analyses were conducted to examine the stability of the main results by constructing two supplementary models: Model 4 excluded participants with extreme exposure values (top and bottom 1.0%) to minimize the effect of outliers, and in Model 5, those with an eGFR below 15 mL/min/1.73 m^2^ were removed from the study cohort, indicative of end-stage renal disease (ESRD), to avoid bias from advanced kidney impairment.

Variance inflation factors (VIF) were calculated to assess multicollinearity among independent variables prior to model fitting. Adjusted generalized VIF values [GVIF^(1/2Df)] were obtained, and a threshold above 5 suggested potential multicollinearity.

Potential nonlinear dose–response relationships were evaluated using restricted cubic spline (RCS) models. The fitted curves were displayed, and *p*-values for overall association and nonlinearity were estimated to detect potential inflection points. To improve interpretability, histograms showing variable distributions were overlaid on the spline plots.

For the hospital-based validation cohort, similar analyses were performed. Given that this cohort represents a clinical non-probability sample, survey weights were not applied. We employed a dichotomization approach for RAR and MAR analogous to that used in NHANES, recalculating ROC curves and optimal cutoff values to enhance comparability and conduct sensitivity assessments. Multivariable logistic regression models in the validation cohort were structured similarly to the NHANES models but included only the available covariates: Model 1 did not include covariate adjustment. Model 2 accounted for age, sex, BMI, smoking status, alcohol consumption, and physical activity. Model 3 additionally incorporated diabetes, hypertension, liver disease, and cancer.

For variables with missing values within the complete-case dataset (<1.0%), multiple imputation using fully conditional specification was performed with the mice package within the R environment. An imputation method based on predictive mean matching was applied for continuous variables (serum creatinine, blood urea nitrogen, MAR, RAR, albumin, RDW, and monocyte counts). Five imputed datasets (m = 5) were generated with 20 iterations each. The imputation model included all exposure variables, outcomes, and covariates used in the primary analyses. Because given the low proportion of missing data, analyses were performed on the first completed dataset for the primary models, with sensitivity analyses yielding consistent results. For subgroup analyses and models susceptible to sparse events or separation, To address small-sample bias in parameter estimates, Firth logistic regression analyses were conducted using the R package logistf.

Causal mediation analysis was conducted using the R package mediation to explore whether hemoglobin levels and the NLR mediated the associations between RAR/MAR and CKD. Three analytic models were used: unadjusted, partially adjusted, and fully adjusted. The average causal mediation effect (ACME) and the average direct effect (ADE) were estimated, and overall effect were obtained through 1,000 bootstrap replications. The mediated proportion was also calculated. To explore potential variation in associations, analyses were stratified by major clinical characteristics, including age, sex, BMI, hypertension, and diabetes. For each subgroup, adjusted odds ratios (ORs) and 95% confidence intervals (CIs) were estimated. Potential interaction effects were assessed using statistical tests. The hospital cohort included only hospitalized patients rather than community-dwelling participants and therefore represented a pathophysiologically distinct group; mediation analyses were not performed for this cohort. Investigations in community-based cohorts will help determine whether the mediation pathways observed in NHANES can be replicated.

The relationships between RAR, MAR, and CKD were evaluated using curve-fitting and threshold analyses. For all analyses, RAR and MAR values were natural log-transformed to normalize distributions, with the primary dataset presented for descriptive purposes.

Statistical analyses were carried out with R (version 4.4.2; R Foundation for Statistical Computing, Vienna, Austria). A two-sided *p* value < 0.05 was considered statistically significant.

## 3 Results

### 3.1 Participant characteristics

Ultimately, 27,072 participants fulfilling the inclusion criteria were analyzed (Figure 1), of whom 13,248 were men (48.9%) and 13,824 were women (51.1%). Participants were classified into CKD and non-CKD groups according to established diagnostic criteria. Of these participants, 4,779 (17.6%) met the criteria for CKD, whereas 22,293 (82.4%) were classified as non-CKD.

Baseline demographic features, lifestyle variables, comorbidities, and laboratory indicators stratified by CKD presence and presented in Table 1. Several variables differed significantly between groups (all *p* < 0.050), accounting for demographic and lifestyle factors such as age, sex, race/ethnicity, education, PIR, smoking, alcohol use, BMI, physical activity, and prior diagnoses of hypertension and diabetes, liver disease, and cancer. CKD prevalence was higher among participants aged ≥50 years (*p* < 0.001) and among those with diabetes, hypertension, or cancer (all *p* < 0.001).

**Table 1:** Baseline characteristics of included participants (*n* = 27072) in the NHANES 2005–2018.

Characteristics	Total	CKD		*p*-value
	*N* = 27072	No (*N* = 22293)	Yes (*N* = 4779)	
Age (%)				<0.001
20–50	14171 (52.3)	13138 (58.9)	1033 (21.6)	
>50	12901 (47.7)	9155 (41.1)	3746 (78.4)	
Gender (%)				0.032
Male	13248 (48.9)	10977 (49.2)	2271 (47.5)	
Female	13824 (51.1)	11316 (50.8)	2508 (52.5)	
Education (%)				<0.001
<High school	2506 (9.3)	1855 (8.3)	651 (13.6)	
Completed high school	3605 (13.3)	2862 (12.8)	743 (15.5)	
>High school	20961 (77.4)	17576 (78.8)	3385 (70.8)	
Race (%)				<0.001
Mexican American	4021 (14.9)	3411 (15.3)	610 (12.8)	
Other Hispanic	2561 (9.5)	2187 (9.8)	374 (7.83)	
Non-Hispanic White	12118 (44.8)	9737 (43.7)	2381 (49.8)	
Non-Hispanic Black	5474 (20.2)	4441 (19.9)	1033 (21.6)	
Other Race	2898 (10.7)	2517 (11.3)	381 (7.9)	
Marriage (%)				<0.001
Married/Living with partner	16342 (60.4)	13705 (61.5)	2637 (55.2)	
Widowed/Divorced/Separated/Never married	10730 (39.6)	8588 (38.5)	2142 (44.8)	
Alcohol (%)	2904 (10.7)	2472 (11.1)	432 (9.0)	<0.001
Smoke (%)				<0.001
Never	15039 (55.6)	12621 (56.6)	2418 (50.6)	
Former	6696 (24.7)	5099 (22.9)	1597 (33.4)	
Current	5337 (19.7)	4573 (20.5)	764 (16.0)	
Sport level (%)				<0.001
Inactive	14324 (52.9)	11419 (51.2%)	2905 (60.8)	
Moderate	6577 (24.3)	5448 (24.4%)	1129 (23.6)	
Vigorous	1276 (4.7)	1119 (5.0)	157 (3.3)	
Both moderate and vigorous	4895 (18.1%)	4307 (19.3%)	588 (12.3)	
PIR (%)				<0.001
≤ 1.00	5592 (20.7)	4549 (20.4)	1043 (21.8)	
1.01–3.00	11300 (41.7)	8998 (40.4)	2302 (48.2)	
>3.00	10180 (37.6)	8746 (39.2)	1434 (30.0)	
BMI (kg/m^2^)				<0.001
Underweight	406 (1.5)	332 (1.5)	74 (1.5)	
Normal	5557 (20.5)	4771 (21.4)	786 (16.4)	
Overweight	7191 (26.6)	6013 (27.0)	1178 (24.6)	
Obesity	13918 (51.4)	11177 (50.1)	2741 (57.4)	
Diabetes (%)	4748 (17.5)	2904 (13.0)	1844 (38.6)	<0.001
Hypertension (%)	9744 (36.0)	6725 (30.2)	3019 (63.2)	<0.001
Liver disease (%)	1060 (3.9)	792 (3.6)	268 (5.6)	<0.001
Cancer (%)	2578 (9.5)	1742 (7.8)	836 (17.5)	<0.001
eGFR (mL·min^−1^·1.73 m^−2^)	93.4 (23.4)	98.2 (18.9)	70.9 (28.9)	<0.001
UACR (mg/g)	43.5 (339.0)	8.09 (5.6)	209 (786.0)	<0.001
MAR	0.13 (0.1)	0.13 (0.1)	0.14 (0.1)	<0.001
RAR	3.17 (0.5)	3.13 (0.5)	3.36 (0.6)	<0.001
Albumin (g/dL)	4.23 (0.3)	4.26 (0.4)	4.13 (0.4)	<0.001
Monocyte (×10^3^ μL^−1^)	0.56 (0.2)	0.55 (0.2)	0.59 (0.3)	<0.001
RDW (%)	13.3 (1.4)	13.2 (1.3)	13.7 (1.6)	<0.001
NLR	2.15 (1.2)	2.07 (1.1)	2.47 (1.5)	<0.001
Hemoglobin (g/L)	141 (15.2)	142 (14.7)	137 (16.8)	<0.001

Mean ± SD was for continuous variables. The percentage (95% confidence interval) was for categorical variables. NHANES, National Health and Nutrition Examination Survey; CKD, chronic kidney disease; PIR, poverty-income ratio; BMI, body mass index; eGFR, estimated glomerular filtration rate; UACR, urine albumin-to-creatinine ratio; RAR, red cell distribution width-to-albumin ratio; MAR, monocyte-to-albumin ratio; NLR, neutrophil-to-lymphocyte ratio; RDW, red blood cell distribution width.

Participants with CKD had elevated MAR and RAR levels relative to participants without CKD (both *p* < 0.001), reflecting greater systemic inflammation and poorer nutritional status. Participants with CKD had reduced serum albumin and hemoglobin levels relative to individuals without CKD (*p* < 0.001), further supporting the potential presence of malnutrition among individuals with CKD.

Furthermore, several immune-inflammatory biomarkers, including the NLR, absolute monocyte count, and RDW, were markedly elevated within the participants diagnosed with CKD (all *p* < 0.001).

Collectively, these results underscore a strong association of CKD and both inflammatory activation and nutritional decline within a nationally representative cohort from the United States at the national level, with demographic, behavioral, and clinical factors influencing this relationship. Table 1 presents a comprehensive comparison of characteristics for participants with and without CKD.

### 3.2 Association between inflammation/nutrition-related indicators and CKD occurrence

Given conceptual overlap and potential collinearity, RAR and MAR were evaluated in separate models. Before model fitting, multicollinearity was evaluated separately in each fully adjusted model. The adjusted VIF values were all close to 1.0 and well below 5, indicating no evidence of multicollinearity (Supplementary Tables 1, 2).

For descriptive stratification purposes, optimal thresholds were identified using the Youden index from unadjusted ROC analyses. The derived cut-off values were 1.419 for RAR and 0.127 for MAR (Supplementary Figures 1A,B). These thresholds were applied to categorize participants into higher and lower biomarker groups. Participants with values above the respective thresholds demonstrated a higher prevalence of CKD relative to those below the thresholds (RAR: 24.6% vs. 12.0%; MAR: 21.8% vs. 14.8%; both *p* < 0.001) (Supplementary Figures 1C,D). These thresholds are data-driven and intended for stratified analyses within the present dataset rather than as clinically validated diagnostic cut-points.

The associations between inflammation- and nutrition-related indices and CKD were examined using multivariable logistic regression models. Elevated RAR and MAR levels showed significant associations with CKD, which persisted after adjustment for known confounders (all *p* < 0.050). In Model 1 (unadjusted), RAR showed a significant association with CKD (OR = 2.39, 95% CI: 2.24–2.55, *p* < 0.001). This association remained in Model 2 after adjustment (OR = 1.81, 95% CI: 1.69–1.94, *p* < 0.001). After further adjustment for hypertension, diabetes, liver disease, and cancer (Model 3), high RAR remained associated with prevalent CKD (OR = 1.68, 95% CI: 1.56–1.80, *p* < 0.001).

In Model 1, MAR levels showed a significant association with CKD (OR = 1.61, 95% CI: 1.51–1.71, *p* < 0.001). After adjustment in Model 2, higher MAR remained associated with prevalent CKD (OR = 1.42, 95% CI: 1.33–1.52, *p* < 0.001). In Model 3, elevated MAR remained associated with prevalent CKD (OR = 1.33, 95% CI: 1.24–1.43, *p* < 0.001) (Table 2).

**Table 2:** Logistic regression analysis for the association between RAR and CKD in various models.

Variable	OR	(95%CI)	*p*-value
RAR			
Model 1			
RAR_low_	Reference	Reference	Reference
RAR_high_	2.39	(2.24–2.55)	<0.001
Model 2			
RAR_low_	Reference	Reference	Reference
RAR_high_	1.81	(1.69–1.94)	<0.001
Model 3			
RAR_low_	Reference	Reference	Reference
RAR_high_	1.67	(1.56–1.80)	<0.001
MAR			
Model 1			
MAR_low_	Reference	Reference	Reference
MAR_high_	1.61	(1.51–1.72)	<0.001
Model 2			
MAR_low_	Reference	Reference	Reference
MAR_high_	1.42	(1.33–1.52)	<0.001
Model 3			
MAR_low_	Reference	Reference	Reference
MAR_high_	1.33	(1.24–1.43)	<0.001

Model 1: crude model, without any adjustments. Model 2: adjusted for age, gender, race, marriage, education, poverty-to-income ratio, body mass index, smoking status, alcohol status and physical activity levels. Model 3: adjusted for all covariates (age, gender, race, marriage, education, poverty-to-income ratio, body mass index, smoking status, alcohol status, physical activity levels, hypertension, diabetes, liver diseases and cancer).

In the hospital-based validation cohort, ROC analyses for RAR and MAR yielded optimal cutoff points of 1.448 and 0.137, respectively, which were comparable to those observed in NHANES (Supplementary Figure 2). In multivariable logistic regression models, CKD was consistently associated with elevated RAR and MAR levels, supporting the robustness of the findings from the primary NHANES cohort. Detailed results for the validation cohort are provided in Supplementary Table 3.

Sensitivity analyses were performed to confirm the robustness of these results (Supplementary Table 4). In Model 4, participants with elevated RAR showed a significant association with CKD (OR = 1.65, 95% CI: 1.53–1.78), as did those with elevated MAR levels (OR = 1.31, 95% CI: 1.22–1.41). In Model 5, participants with higher RAR levels again showed an elevated probability of CKD (OR = 1.65, 95% CI: 1.54–1.78), and similarly, participants with elevated MAR showed a significant association with CKD (OR = 1.33, 95% CI: 1.24–1.42; Supplementary Table 4). These multivariable logistic regression analyses underscore that both RAR and MAR are strongly related to CKD, showing that these composite indices may offer value in characterizing CKD status within this cross-sectional population.

For the hospital-based validation cohort, we performed sensitivity analyses using the same logistic regression models as in the NHANES analyses, including adjustments for demographic, lifestyle, and clinical covariates. The findings aligned with the primary NHANES results, with both RAR and MAR showing positive associations with prevalent CKD (Supplementary Table 5), supporting the robustness of the observed relationships in an independent clinical population.

### 3.3 Nonlinear analysis of inflammation/nutrition-related indicators and CKD occurrence

**Figure 2:**
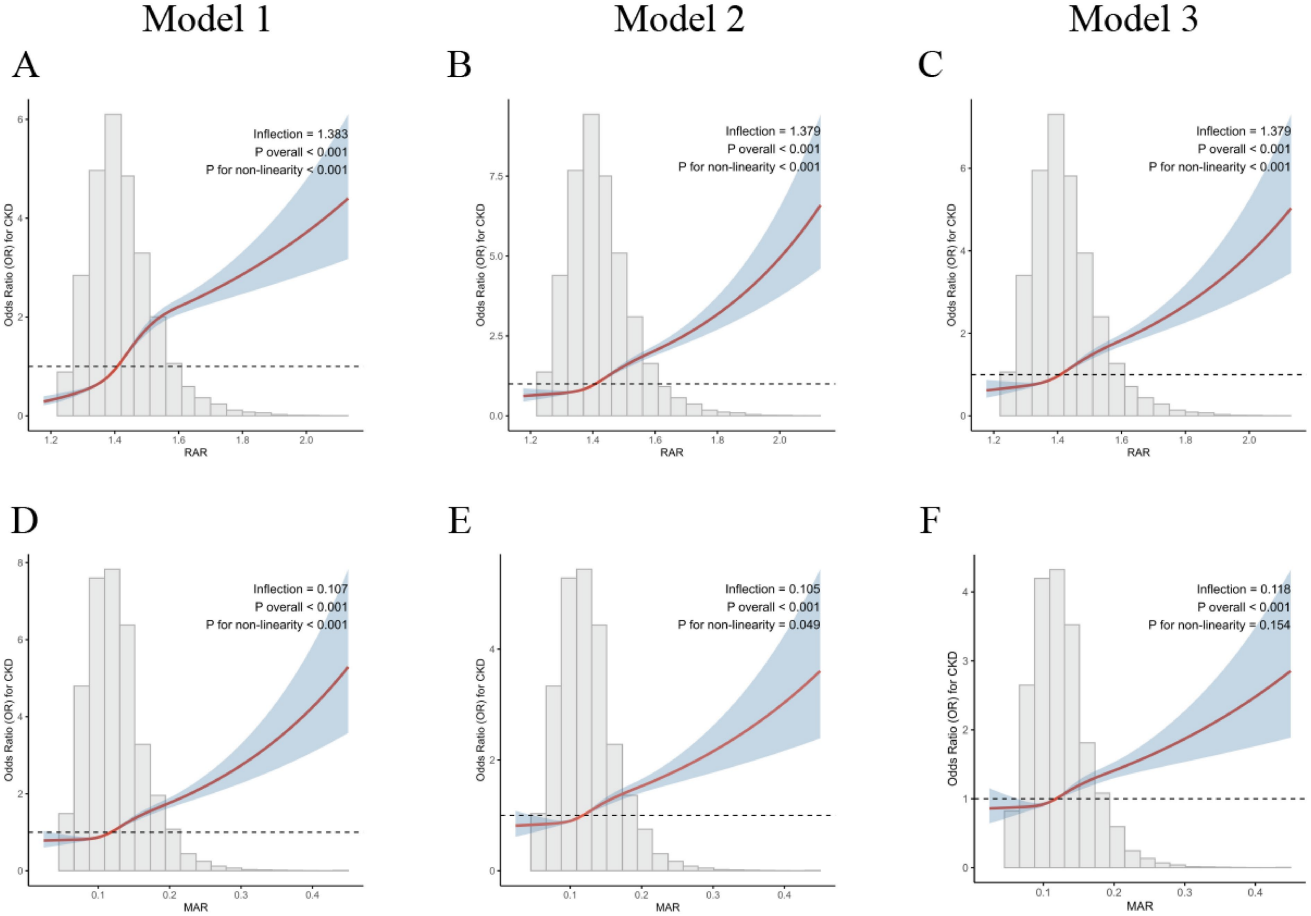
The dose–response association between the inflammation/nutrition-related indicators and CKD. After adjusting for covariates, we used a restricted cubic spline (RCS) regression model to evaluate the association between inflammation/nutrition-based indicators and CKD. In the figure, the red solid line represents the estimated central tendency of the association, while the blue shaded area indicates the 95% confidence interval. Model 1: crude model, without any adjustments. Model 2: adjusted for age, gender, race, marriage, education, poverty-to-income ratio, body mass index, smoking status, alcohol status. Model 3: adjusted for all covariates (age, gender, race, marriage, education, poverty-to-income ratio, body mass index, smoking status, alcohol status, hypertension, diabetes, liver diseases, cancer). **(A–C)** Nonlinear association between RAR and CKD in Models 1–3, respectively. **(D–F)** Nonlinear association between MAR and CKD in Models 1–3, respectively.

**Figure 3:**
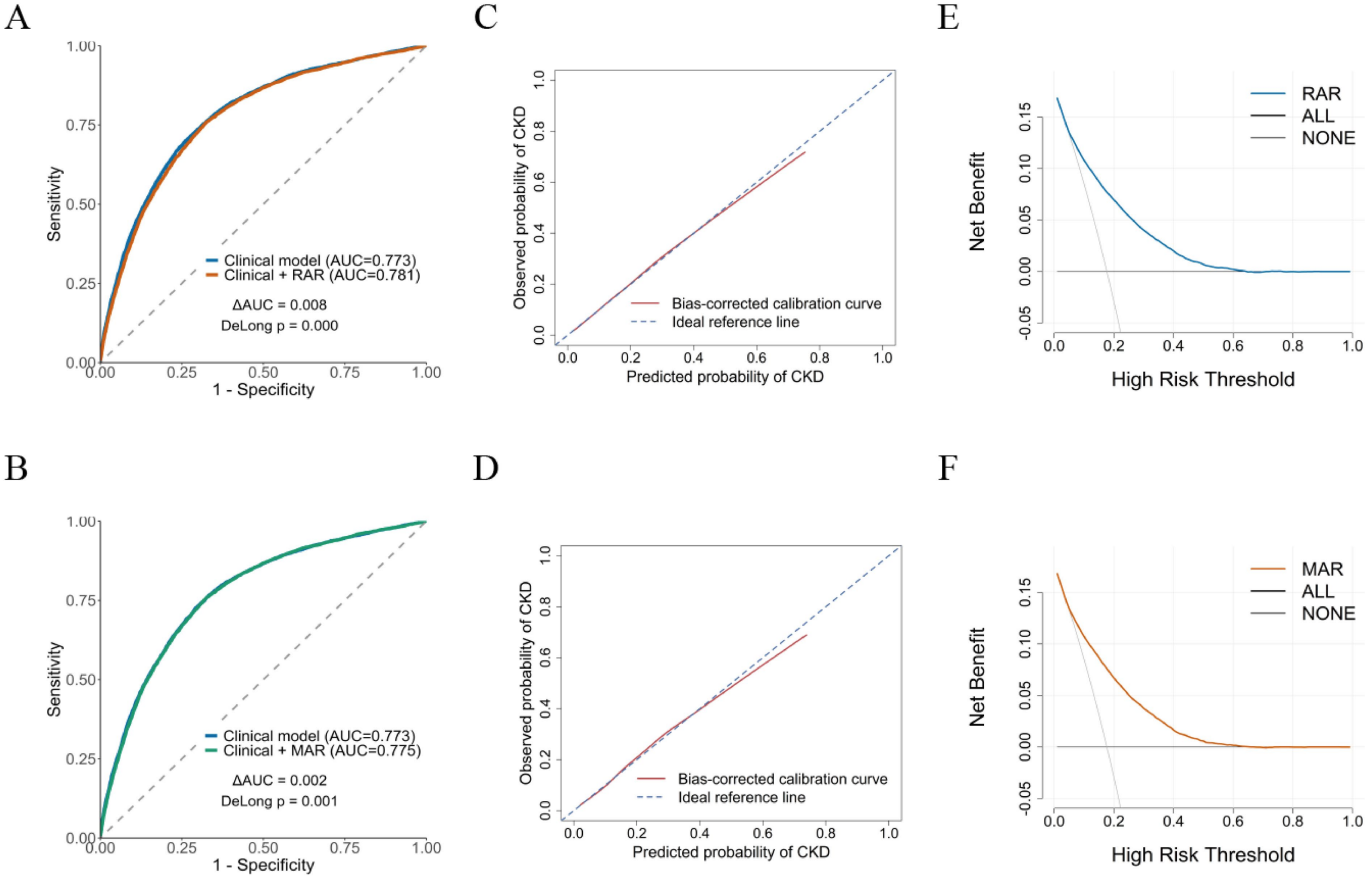
Discriminatory performance, calibration, and decision curve analysis of clinical models incorporating inflammation/nutrition-related indicators for CKD identification. Receiver operating characteristic (ROC) curves were used to evaluate the discriminatory ability of the clinical model and models incorporating RAR or MAR **(A,B)**. Calibration plots were used to assess the agreement between estimated and observed probabilities of CKD **(C,D)**. Decision curve analysis (DCA) was performed to evaluate the potential net clinical benefit of the models across a range of threshold probabilities **(E,F)**. In the calibration plots, the diagonal dashed line represents perfect agreement between estimated and observed risks, while the solid curve represents the model-estimated calibration. In the decision curves, the horizontal line indicates the “treat-none” strategy and the slanted line indicates the “treat-all” strategy.

To better describe the dose–response relationship between RAR, MAR, and CKD, restricted cubic spline terms were included in multivariable logistic regression models to assess potential nonlinear relationships. The results are displayed in Figure 2. For RAR (Figure 2A), a nonlinear association was observed in the basic analyses (Model 1; *P* for nonlinearity < 0.001). In Models 2 (Figure 2B) and 3 (Figure 2C), which included additional health-related covariates, a nonlinear relationship between RAR and CKD was observed (*P* for nonlinearity < 0.001). These findings indicate a nonlinear relationship between RAR levels and CKD.

In Model 1 (Figure 2D), MAR showed a significant nonlinear association (*P* for nonlinearity < 0.001). However, the nonlinear trend weakened in Model 2 (Figure 2E) (*p* for nonlinearity = 0.049). After full adjustment (Model 3; Figure 2F), the nonlinear relationship was no longer evident (*p* for nonlinearity = 0.154), with the relationship between MAR levels and CKD appearing predominantly linear.

In the hospital-based validation cohort, restricted cubic spline analyses were conducted following the same methodology as in the NHANES dataset. Similar dose–response trends for RAR and MAR in relation to CKD risk were observed in the validation cohort, with estimated odds ratios following patterns comparable to those in the main NHANES analysis (Supplementary Figure 3).

Despite this change, the overall trend remained positive, with higher MAR levels linked to higher CKD occurrence. Overall, MAR could serve as a composite marker of inflammation and nutritional status among individuals with CKD.

### 3.4 Discrimination, calibration, and decision-analytic evaluation of the clinical models

The diagnostic performance of the biomarkers and the models’ discriminative ability were examined using ROC curve analysis. In models including only the biomarkers (Supplementary Figures 1A,B), the AUC for RAR increased from 0.644 (95% CI: 0.64–0.65) in Model 1 to 0.781 (95% CI: 0.77–0.79) in the fully adjusted Model 3 (Supplementary Table 6). Similarly, the AUC for MAR levels rose from 0.577 (95% CI: 0.57–0.58) to 0.775 (95% CI: 0.77–0.78) after full adjustment, indicating improved discriminative ability with the inclusion of demographic and clinical covariates.

Receiver operating characteristic analyses showed that the clinical model demonstrated an AUC of 0.773 for CKD identification (Figures 3A,B). The addition of RAR resulted in a modest improvement in discrimination (AUC = 0.781; ΔAUC = 0.008; DeLong *p* < 0.001). In contrast, incorporation of MAR yielded only a minimal increase in AUC (AUC = 0.775; ΔAUC = 0.002; DeLong *p* = 0.001; Supplementary Table 7). RAR exhibited a marginally higher ability to discriminate CKD status than MAR when incorporated into conventional clinical models.

Calibration plots showed good agreement between predicted and observed CKD prevalence (Figures 3C,D). For the RAR-based model, the C-index was 0.778 and the Brier score was 0.122; for the MAR-based model, the C-index was 0.774 and the Brier score was 0.123. Both models had calibration intercepts near zero and slopes around 1, suggesting minimal systematic error and well-calibrated predicted probabilities.

Both models exhibited net benefit across the evaluated thresholds, as shown by decision curve analysis (Figures 3E,F). In the 10–30% threshold range, the RAR model achieved a mean adjusted net benefit of 0.002, reaching a peak of 0.004 at a threshold probability near 0.27. The MAR model exhibited a smaller mean net benefit of 0.001, reaching a maximum near a threshold probability of 0.29.

Performance analyses indicated that both biomarkers contributed to the ability of clinical variables to discriminate individuals with CKD. Adding RAR resulted in a modestly larger enhancement in model discrimination compared with MAR. The models demonstrated satisfactory calibration, with predicted probabilities closely aligning with observed outcomes. Decision curve analysis showed that both models provided net benefit across the assessed thresholds, with the RAR-based model providing a slightly higher benefit.

### 3.5 Subgroup analysis

**Figure 4:**
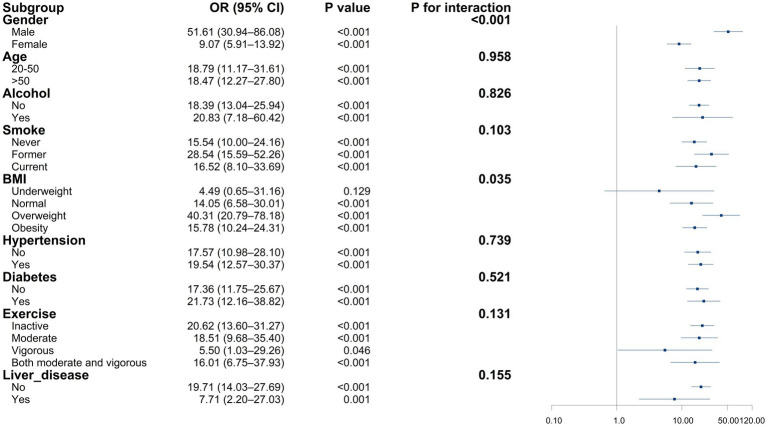
Association between RAR and CKD in subgroup and interactive analyses. The relationship between inflammation/nutrition-based indicators and CKD based on subgroups. Forest plot of the relationship of CKD with RAR in different subgroups. Adjusted for age, gender, race, marriage, education, poverty-to-income ratio, body mass index, smoking status, alcohol status, hypertension, diabetes, liver diseases, cancer.

The relationship between RAR and CKD varied across population subgroups (Figure 4). After stratification by sex, age, alcohol use, smoking status, BMI, hypertension, diabetes, physical activity, and liver disease, RAR remained significantly associated with CKD in all subgroups (all *p* < 0.050). Interaction analysis indicated a significant interaction between sex and RAR (*P* for interaction < 0.001), with the relationship between RAR levels and CKD varying by sex. A significant interaction between BMI and RAR was observed (*P* for interaction = 0.035). In the underweight group, the relationship between RAR levels and CKD did not reach statistical significance (OR = 4.49, 95% CI: 0.65–31.16, *p* = 0.129), but a positive association persisted in the other BMI categories. Apart from sex and BMI, no other stratified variables showed significant interactions, indicating limited impact on the RAR–CKD relationship.

**Figure 5:**
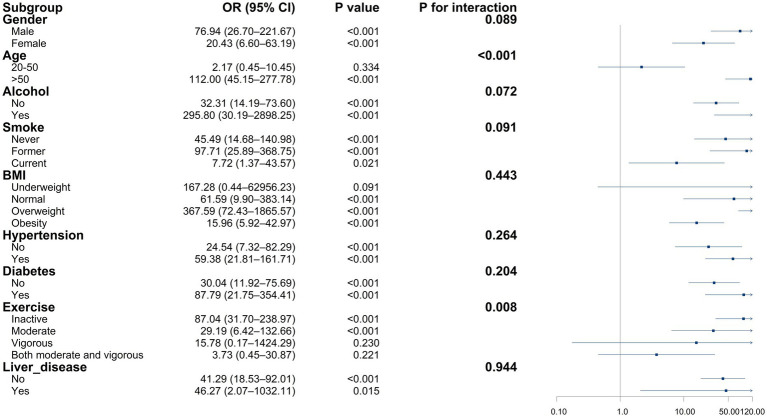
Association between MAR and CKD in subgroup and interactive analyses. The relationship between inflammation/nutrition-based indicators and CKD based on subgroups. Forest plot of the relationship of CKD with MAR in different subgroups. Adjusted for age, gender, race, marriage, education, poverty-to-income ratio, body mass index, smoking status, alcohol status, hypertension, diabetes, liver diseases, cancer.

Interaction analysis revealed that age significantly influenced the relationship between MAR levels and CKD (*P* for interaction < 0.001; Figure 5). In the 20–50 age group, MAR showed no significant association with CKD (OR = 2.17, 95% CI: 0.45–10.45, *p* = 0.334). Apart from age, no other variables demonstrated significant interactions, and no apparent effect modification on the MAR–CKD association was observed.

In the hospital-based validation cohort, subgroup analyses followed the same methodology as in the NHANES dataset. The patterns observed for RAR and MAR aligned with the primary NHANES findings (Supplementary Figure 4). A significant age and MAR interaction was observed within the hospital cohort (interaction *p* < 0.001), consistent with the NHANES results. For RAR, the previously observed interaction with sex remained evident; however, the significant interaction between BMI and RAR seen in the NHANES cohort did not hold within the using data from validation cohort (*p* > 0.050). These findings suggest that the direction and magnitude of certain subgroup associations may vary between community-based and hospital-based populations, potentially reflecting differences in clinical characteristics or sample composition.

### 3.6 Mediation analysis

**Figure 6:**
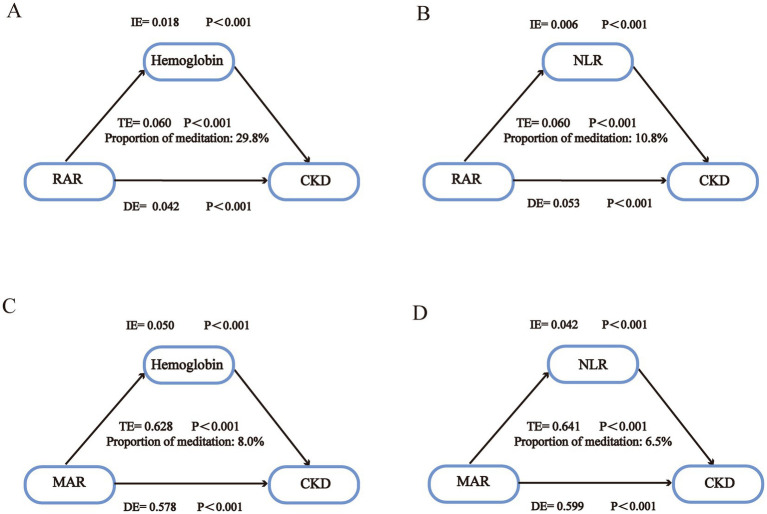
Mediation analysis of the interaction between hemoglobin and NLR levels on three indices of inflammation/nutrition-related indicators and the risk of CKD. Mediation effects of hemoglobin and NLR on the associations between RAR/MAR and CKD prevalence [Panels **(A–D)** represent hemoglobin **(A,C)** and NLR **(B,D)** as mediators for RAR and MAR, respectively]. IE, Indirect effect. DE, Direct effect. TE, Total effect. NLR, neutrophil-to-lymphocyte ratio.

As shown in Figure 6A, controlling for major covariates including age, sex, BMI, race/ethnicity, education, lifestyle factors, and clinical comorbidities, hemoglobin exerted a significant indirect effect on the relationship between RAR levels and CKD. The average causal mediation effect (ACME) was 0.018 (95% CI: 0.01–0.02, *p* < 0.001), accounting for 29.8% of the total effect. Similarly, NLR also showed a significant indirect effect within the same statistical mediation model, with an ACME of 0.006 (95% CI: 0.00–0.01, *p* < 0.001), contributing 10.8% to the overall impact (Figure 6B) (32, 33).

Subgroup analyses (Figures 4, 5) revealed a significant interaction between RAR and sex. To further explore whether the statistical mediation patterns differed by sex, stratified mediation analyses were conducted. In both males and females, the statistically estimated mediation effect of hemoglobin remained significant. In males, hemoglobin statistically accounted for 26.9% of the total association (ACME = 0.012, 95% CI: 0.01–0.02, *p* < 0.001), while in females, the proportion statistically accounted for was 31.1% (ACME = 0.022, 95% CI: 0.01–0.03, *p* < 0.001). These results suggest that sex may influence the magnitude of the statistical mediation effect of hemoglobin in the RAR–CKD association, potentially due to differences in physiological or clinical factors. For NLR, the mediation effect was 10.2% in males (ACME = 0.005, *p* < 0.001) and 9.7% in females (ACME = 0.007, *p* < 0.001), indicating comparable statistical mediation patterns across sexes. Given the cross-sectional nature of the study, the observed associations cannot be interpreted as evidence of causation.

In the MAR analysis (Figures 6C, D), hemoglobin showed a significant indirect effect after controlling for all relevant covariates, yielding an ACME of 0.050 (95% CI: 0.03–0.07, *p* < 0.001), accounting for 8.0% of the total effect. NLR also showed a significant mediating effect, with an ACME of 0.042 (95% CI: 0.03–0.06, *p* < 0.001), corresponding to 6.5% of the total effect (95% CI: 3.9–10.0%). These results indicate that hemoglobin and NLR explained a fraction of the association observed between MAR and CKD prevalence. Detailed results for RAR and MAR models are presented in Supplementary Tables 8, 9.

## 4 Discussion

This cross-sectional analysis of 27,072 NHANES participants representative of the U. S. population found positive associations between the red cell distribution width-to-albumin ratio (RAR), the monocyte-to-albumin ratio (MAR), and CKD prevalence. These associations were consistent across subgroups. Interaction and mediation analyses suggested that sex may modify the relationship of RAR with CKD. Given the cross-sectional design, the findings reflect associations and do not imply causality or temporal sequence.

The kidneys play an essential role in immunoregulation and systemic immune homeostasis, but they are vulnerable to inflammatory injury during CKD progression. Chronic inflammation and related pathophysiological mechanisms can lead to tubular injury, which is associated with CKD progression (34, 35). Cytokines with pro-inflammatory activity, including interleukins and TNF-*α*, are capable of activating endothelial cells and promoting monocyte recruitment. Elevated RDW may be related to inflammatory activity, potentially through alterations in red blood cell integrity or hematopoiesis. Nutritional impairment often coexists with inflammation, and both processes may be associated with renal function decline (36).

Förhécz et al. reported associations of RDW with indicators of inflammation and nutritional profile, and renal function (37) Monocytes, key markers of chronic inflammation, have been shown by Jiang et al. to be positively correlated with the emergence and advancement of acute kidney injury (AKI) (38). Additionally, serum albumin is widely recognized as a robust biomarker capable of reflecting both inflammatory and nutritional condition (39). The inflammation/nutrition indices used in this study combine biomarkers of inflammation and nutrition. RAR and MAR were associated with prevalent CKD in this analysis. Previous research has suggested that RAR and MAR are associated with renal outcomes and may have potential clinical relevance (18, 40, 41). Prior investigations have indicated a significant link between RAR and the incidence of diabetic kidney disease (DKD) (42), as well as with CKD prognosis (19). The monocyte-to-lymphocyte ratios have been related to mortality within a cohort of type 2 diabetes patients with CKD (43). However, evidence regarding the link of MAR with CKD is limited.

Further analysis evaluated the association of RAR with CKD differed by sex. Mediation analyses suggested that hemoglobin and NLR showed a significant association with CKD prevalence in relation to composite inflammation and nutrition indices, and the association involving hemoglobin appeared stronger among women compared with men. These observations could reflect biological differences specific to sex, including hormonal factors. Estrogen has been associated with increased expression of proteins involved in renal protection, whereas testosterone has been associated with less favorable renal profiles in some studies (44, 45). Given the cross-sectional design, the temporal sequence among exposure, mediator, and outcome cannot be established. Accordingly, the mediation outcomes ought to regard as preliminary and not considered conclusive evidence of causal pathways. Longitudinal research is warranted to clarify whether hemoglobin and NLR are involved in the relationship between indices related to inflammation and nutritional status and CKD. These findings suggest possible sex-related differences in CKD and warrant further investigation.

Compared with conventional inflammatory biomarkers, RAR and MAR are composite indices that combine measures of inflammation and nutrition. Traditional markers such as TNF-*α* and interleukins may be less practical in routine clinical settings because of higher costs and greater testing complexity. In contrast, RAR and MAR are calculated from routinely collected laboratory parameters. Associations involving RAR and MAR have been reported in various disease contexts, including CKD (18, 19, 21, 42). Their availability in routine laboratory testing may facilitate their use in examining associations with CKD across different healthcare settings.

However, these associations should be interpreted with caution because of possible residual confounding. Although multivariable adjustments were performed, detailed data on CKD-related medication use were not available. Pharmacological treatments, such as renin–angiotensin–aldosterone system (RAAS) blockers and SGLT2 inhibitors, statins, anti-inflammatory agents, erythropoiesis-stimulating agents, and iron supplementation may affect inflammatory markers or hematologic parameters, which could in turn influence the calculated indices and CKD classification (46, 47). Acute infections or transient inflammatory conditions may not have been fully excluded. In addition, serum albumin concentrations may be influenced by factors unrelated to nutritional status, including hydration status and underlying liver function, which could introduce additional variability in the calculated ratios. Because RAR and MAR were derived from single-time-point hematologic and biochemical measurements, they may reflect acute inflammatory responses rather than chronic low-grade inflammation associated with CKD, which could contribute to measurement variability.

In individuals with prolonged disease or advanced CKD, malnutrition and chronic low-grade inflammation are more common and have been associated with factors such as anorexia, viral gastritis, and the accumulation of uremic toxins (48). These factors have been linked to the malnutrition-inflammation syndrome, which is characterized by lower albumin and hemoglobin levels and higher inflammatory cell counts (49). Complications of advanced CKD, such as cardiovascular disease, have been associated with alterations in peripheral WBC counts (50). Among patients with diabetic CKD, treatments such as glucose-lowering therapies, insulin, and other medications may be associated with changes in serum albumin or peripheral WBC counts (51–53). Longitudinal research is warranted to consider these factors and investigate temporal relationships.

This study has several strengths. It examined associations between inflammation/nutrition-related indices and CKD in a large sample. Analysis of a nationally representative NHANES dataset supports the applicability of the results to the U. S. adults residing in the community. Adjustment for multiple covariates helped address potential confounding. Subgroup analyses showed similar associations across key strata. An independent hospital-based cohort based at the Fifth Affiliated Hospital of Sun Yat-sen University was used for replication analyses. ROC and multivariable logistic regression analyses in this cohort yielded patterns similar to those observed in NHANES (see Supplementary material).

The study also has several limitations. First, the analysis employed a cross-sectional design, which precludes determination of causality. Although multiple confounders were adjusted for, unmeasured factors such as CKD duration, complication severity, medication use, and acute infections may still influence the observed associations. Serum albumin functions as a negative acute-phase protein, which may be influenced by systemic inflammation and hydration levels, liver function, and overall nutritional status, which may influence the calculated RAR and MAR values. Second, some variables in NHANES were obtained from participant reports and might be affected by potential recall bias. Third, the validation cohort was derived from a hospital clinical population rather than a probability sample, and may include individuals with more advanced or treatment modified disease than the NHANES population. Because of these characteristics, mediation analyses could not be reliably performed in this cohort. Finally, external validity should be considered. NHANES represents the community dwelling population of the United States and might not accurately represent populations in other nations with varying ethnic backgrounds and dietary habits, or health care systems.

Studies incorporating institutionalized and populations with greater clinical severity should be studied to assess the generalizability of these findings. Future prospective multicenter studies with larger sample sizes and longer follow-up should be conducted to examine these associations longitudinally, clarify temporal relationships, and explore potential mediating pathways underlying the link of inflammatory and nutritional indicators to CKD.

## 5 Conclusion

Overall, our findings suggest a strong relationship between indices related to inflammation and nutritional status, specifically RAR and MAR, and CKD. The results from the hospital-based validation cohort corroborated the findings derived from the NHANES analyses. These results suggest that RAR and MAR could be valuable indicators for detecting and intervention of CKD, with hemoglobin and NLR identified as potential mediators of these associations. This research offers novel insights into how inflammatory and nutritional variables relate to CKD; however, given the cross-sectional design, these results should be viewed as evidence of association rather than causality, and further prospective research is necessary to investigate temporal and causal links.

## Data Availability

Data for this study were derived from the National Health and Nutrition Examination Survey (NHANES), a publicly accessible database (https://www.cdc.gov/nchs/nhanes/). Data from the validation cohort are provided in the main text and Supplementary materials. Additional information is available from the corresponding author upon reasonable request.

## Footnotes

1
https://www.cdc.gov/nchs/nhanes/Default.aspx
