# Supplementary material for: Associations of inflammation/nutrition-related indicators (RAR and MAR) with chronic kidney disease: evidence from NHANES 2005–2018
Source: Front Nutr. 2026 Apr 27;13:1725996. doi: 10.3389/fnut.2026.1725996 (PMC13159209; doi:10.3389/fnut.2026.1725996)
Supplement: Supplementary file 1 [file Data_Sheet_1.docx]

**Supplementary material**

# **Supplementary Figure 1**. ROC analysis of RAR and MAR for discrimination of prevalent CKD


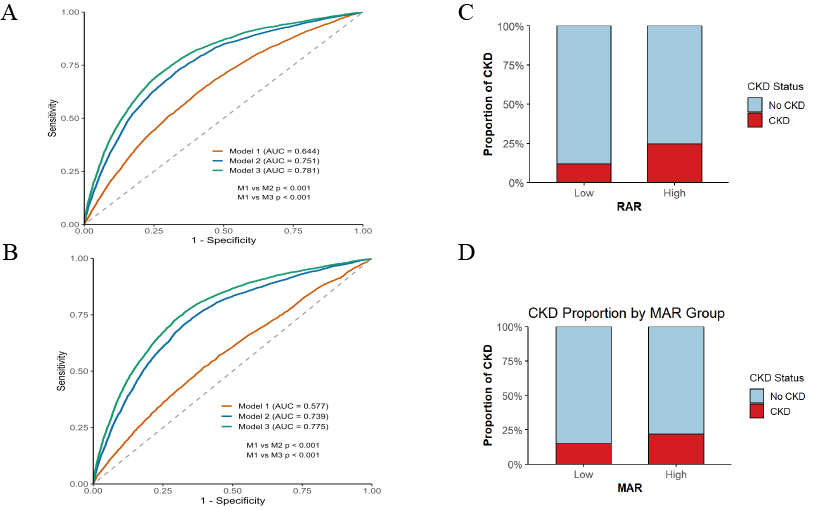


(A, B) Receiver operating characteristic (ROC) curve analyses used to determine optimal cut-off values for RAR and MAR based on the Youden index. The identified thresholds were 1.419 for RAR and 0.127 for MAR. (C, D) Prevalence of CKD according to the categorized RAR and MAR levels based on the derived thresholds. Participants with values above the cut-offs showed a higher prevalence of CKD compared with those below the thresholds.

**Supplementary Figure 2.** ROC analysis of the association between inflammatory nutritional indicators and CKD in different models using hospital case data


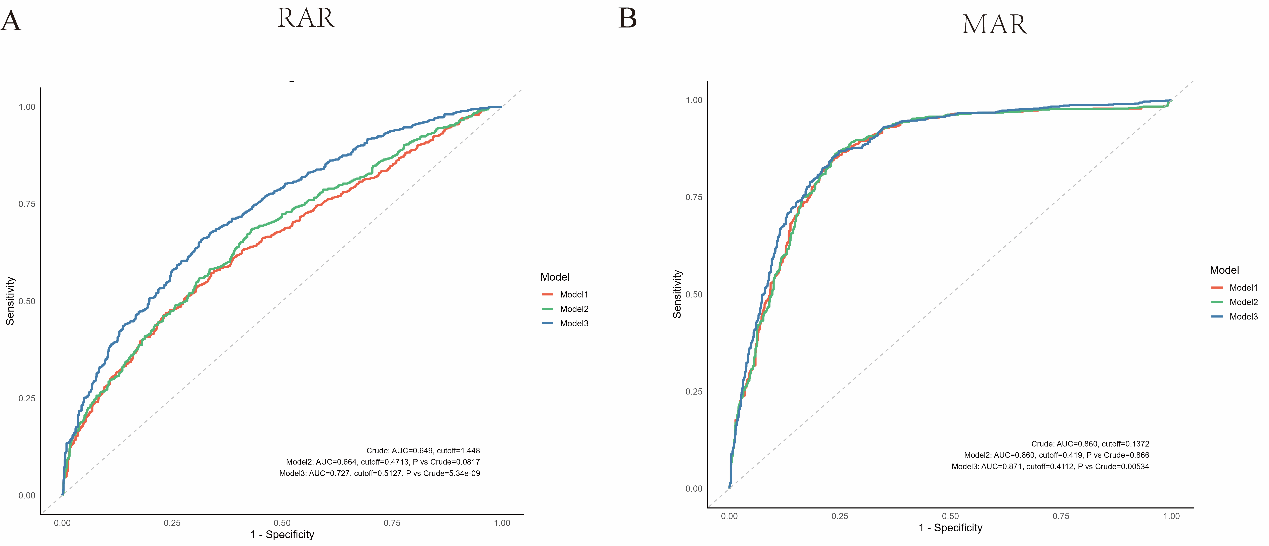


cutoff determination. A: The cutoff of RAR for the prediction of CKD elevated by receiver operating characteristic curve(AUC = 0.649, cutoff=1.448, p<0.001). B: The cutoff of MAR for the prediction of CKD elevated by receiver operating characteristic curve(AUC = 0.86, cutoff=0.137 p<0.001). RAR, red cell distribution width-to-albumin ratio; MAR, monocyte-to-albumin ratio; AUC, Area under curve.

For comparability, we present two sets of NHANES-adjusted estimates: (1) models adjusted for the full set of covariates (age, gender, race/ethnicity, marital status, education, PIR, BMI, smoking, alcohol, hypertension, diabetes, liver disease, cancer), and (2) models adjusted for the reduced covariate set used in the hospital validation cohort (age, gender, BMI, smoking, alcohol, hypertension, diabetes, liver disease, cancer). The effect estimates obtained using the reduced covariate set were broadly consistent in direction with those from the fully adjusted NHANES models, although point estimates and confidence intervals differed modestly (see Supplementary Table5), indicating that differences in socioeconomic covariates may partially account for observed effect-size differences between cohorts.

# **Supplementary Figure 3.** RCS analyses of the associations of RAR and MAR with CKD risk in the hospital-based validation cohort.


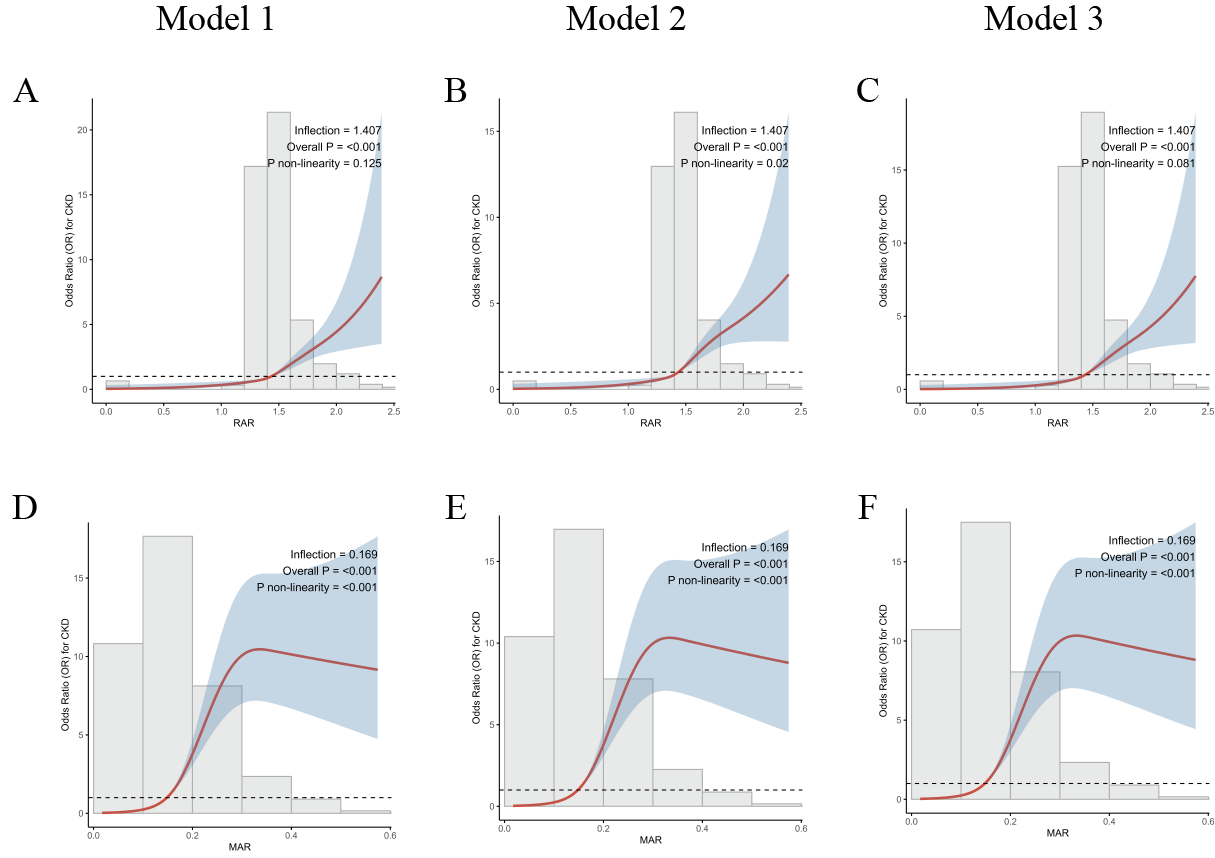


Panels A–C present the associations between RAR and CKD across Model 1 (A), Model 2 (B), and Model 3 (C), while panels D–F present the corresponding associations for MAR across Model 1 (D), Model 2 (E), and Model 3 (F). The red solid lines represent the estimated associations, and the shaded areas indicate 95% confidence intervals. Model 1 is unadjusted. Model 2 is adjusted for age, sex, body mass index, smoking status, and alcohol consumption. Model 3 is further adjusted for hypertension, diabetes, liver disease, and cancer.

**Supplementary Figure 4**. Subgroup analyses of RAR and MAR with CKD risk in the hospital-based validation cohort


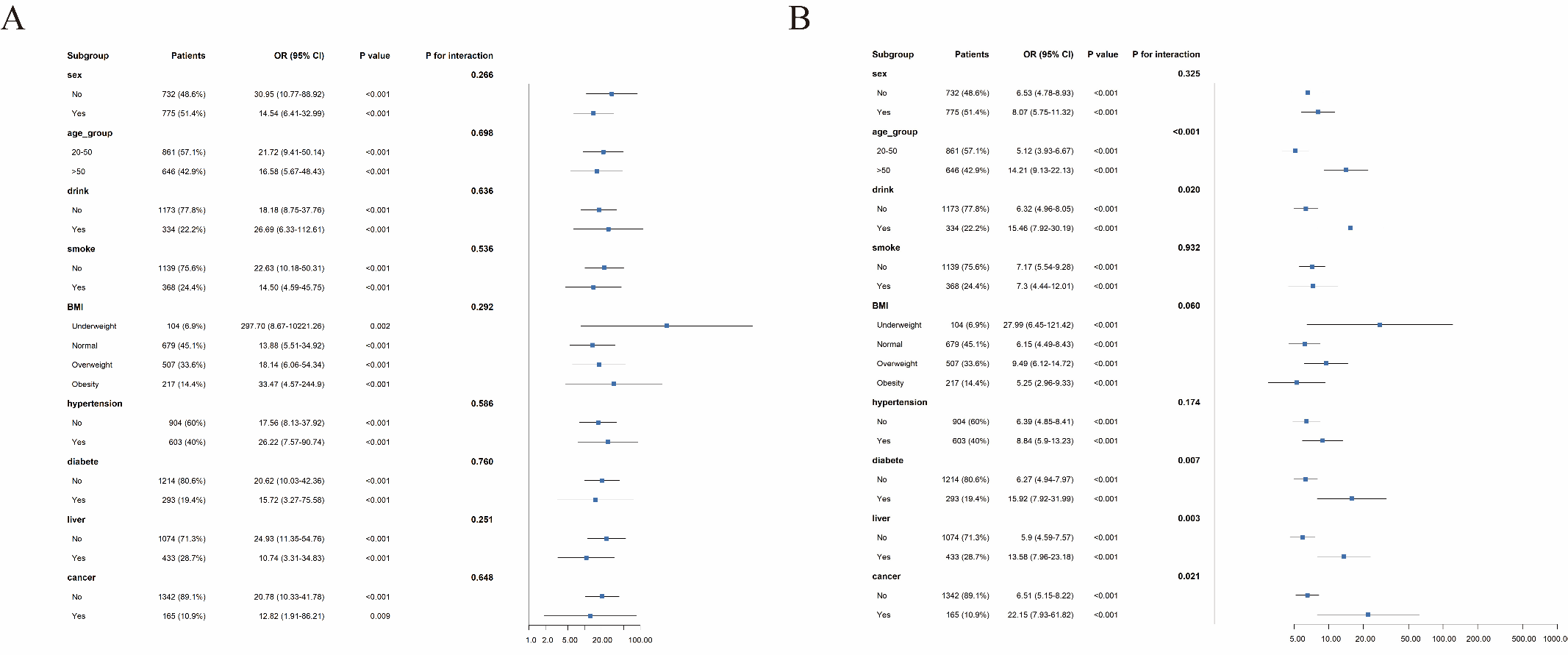


The relationship between inflammation/nutrition-based indicators and CKD based on subgroups. A: Forest plot of the relationship of CKD with RAR in different subgroups. B: Forest plot of the relationship of CKD with MAR in different subgroups. Adjusted for age, gender, body mass index, smoking status, alcohol status, hypertension, diabetes, liver diseases, cancer)

# **Supplementary Table 1** Multicollinearity assessment (VIF) of RAR.

| **Variable** | **VIF** |
| --- | --- |
| RAR | 1.070 |
| Age | 1.117 |
| Gender | 1.076 |
| Education | 1.075 |
| Race | 1.051 |
| Marital | 1.058 |
| Alcohol | 1.032 |
| Smoke | 1.049 |
| Sport level | 1.013 |
| PIR | 1.060 |
| BMI | 1.189 |
| Diabetes | 1.077 |
| Hypertension | 1.093 |
| Liver disease | 1.009 |
| Cancer | 1.039 |

# **Supplementary Table 2** Multicollinearity assessment (VIF) of MAR**.**

| **Variable** | **VIF** |
| --- | --- |
| MAR | 1.033 |
| Age | 1.118 |
| Gender | 1.069 |
| Education | 1.075 |
| Race | 1.047 |
| Marital | 1.058 |
| Alcohol | 1.031 |
| Smoke | 1.050 |
| Sport level | 1.013 |
| PIR | 1.060 |
| BMI | 1.178 |
| Diabetes | 1.076 |
| Hypertension | 1.097 |
| Liver disease | 1.008 |
| Cancer | 1.038 |

# **Supplementary Table 3** Logistic regression analysis of the association between RAR and CKD in different models using hospital case data

| **Variable** | **OR** | **(95%CI)** | **P-value** |
| --- | --- | --- | --- |
| RAR | | | |
| Model 1 | | | |
| RAR_low_ | reference | reference | reference |
| RAR_high_ | 2.59 | (2.10 - 3.19) | <0.001 |
| Model 2 | | | |
| RAR_low_ | reference | reference | reference |
| RAR_high_ | 2.79 | (2.25 - 3.45) | <0.001 |
| Model 3 | | | |
| RAR_low_ | reference | reference | reference |
| RAR_high_ | 2.74 | (2.19 - 3.43) | <0.001 |
| MAR  Model 1 | | | |
| Model 1 | | | |
| MAR_low_ | reference | reference | reference |
| MAR_high_ | 18.17 | (14.01 - 23.58) | <0.001 |
| Model 2 | | | |
| MAR_low_ | reference | reference | reference |
| MAR_high_ | 18.22 | (14.00 - 23.70) | <0.001 |
| Model 3 | | | |
| MAR_low_ | reference | reference | reference |
| MAR_high_ | 18.55 | (14.07 - 24.45) | <0.001 |

Model 1: crude model, without any adjustments..

Model 2: adjusted for age, gender, body mass index, smoking status, alcohol status.

Model 3: adjusted for all covariates (age, gender, body mass index, smoking status, alcohol status, hypertension, diabetes, liver diseases, cancer)

**Supplementary Table 4.** Sensitivity analysis for the association between inflammation/nutrition-based indicators and CKD in various models.

| **Variable** | **OR** | **(95%CI)** | **P-value** |
| --- | --- | --- | --- |
| RAR | | | |
| Model 4 | | | |
| RAR_low_ | reference | reference | reference |
| RAR_high_ | 1.65 | (1.53 - 1.78) | <0.001 |
| Model 5 | | | |
| RAR_low_ | reference | reference | reference |
| RAR_high_ | 1.65 | (1.54 - 1.78) | <0.001 |
| MAR | | | |
| Model 4 | | | |
| MAR_low_ | reference | reference | reference |
| MAR_high_ | 1.31 | (1.22 - 1.41) | <0.001 |
| Model 5 | | | |
| MAR_low_ | reference | reference | reference |
| MAR_high_ | 1.32 | (1.24- 1.42) | <0.001 |

**Model 4:** Model 1 + excluding participants with extreme values of the exposure variables (top and bottom 1%). **Model 5:** Model 1 + excluding participants with an estimated glomerular filtration rate (eGFR) <15 mL/min/1.73 m², corresponding to end-stage renal disease (ESRD).

# **Supplementary Table 5**. Sensitivity analysis of the association between inflammatory/nutritional indicators and CKD in different models using hospital case data

| **Variable** | **OR** | **(95%CI)** | **P-value** |
| --- | --- | --- | --- |
| RAR | | | |
| Model 1 | | | |
| RAR_low_ | reference | reference | reference |
| RAR_high_ | 2.72 | (2.18 - 3.40) | <0.001 |
| Model 2 | | | |
| RAR_low_ | reference | reference | reference |
| RAR_high_ | 2.63 | (2.11 - 3.30) | <0.001 |
| Model 3 | | | |
| RAR_low_ | reference | reference | reference |
| RAR_high_ | 2.49 | (1.98 - 3.14) | <0.001 |
| MAR | | | |
| Model 1 | | | |
| MAR_low_ | reference | reference | reference |
| MAR_high_ | 18.55 | (14.14 - 24.57) | <0.001 |
| Model 2 | | | |
| MAR_low_ | reference | reference | reference |
| MAR_high_ | 19.95 | (15.10 - 26.62) | <0.001 |
| Model 3 | | | |
| MAR_low_ | reference | reference | reference |
| MAR_high_ | 18.89 | (14.29 - 25.21) | <0.001 |

Model 1: adjusted for all covariates (age, gender, body mass index, smoking status, alcohol status, hypertension, diabetes, liver diseases, cancer).

Model 2: Model 1 + excluding participants with extreme values of the exposure variables (top and bottom 1%)

Model 3: Model 1 + excluding participants with an estimated glomerular filtration rate (eGFR) <15 mL/min/1.73 m², corresponding to end-stage renal disease (ESRD).

**Supplementary Table 6** Discriminatory performance of MAR and RAR models for CKD

| **Model** | **AUC (95% CI)** | **Cut-off** | **Sensitivity** | **Specificity** |
| --- | --- | --- | --- | --- |
| RAR | | | | |
| Model 1 | 0.644 (0.64-0.65) | 1.419 | 0.626 | 0.589 |
| Model 2 | 0.751 (0.74-0.76) | 0.193 | 0.727 | 0.663 |
| Model 3 | 0.781 (0.77-0.79) | 0.195 | 0.701 | 0.738 |
| MAR | | | | |
| Model 1 | 0.577 (0.57-0.59) | 0.127 | 0.516 | 0.605 |
| Model 2 | 0. 739 (0.73-0.75) | 0.206 | 0.707 | 0.674 |
| Model 3 | 0. 775 (0.77-0.78) | 0.176 | 0.726 | 0.708 |

**Model 1:** crude model, without any adjustments. **Model 2:** adjusted for age, gender, race, marriage, education, poverty-to-income ratio, body mass index, smoking status, alcohol status and physical activity levels. **Model 3:** adjusted for all covariates (age, gender, race, marriage, education, poverty-to-income ratio, body mass index, smoking status, alcohol status, physical activity levels, hypertension, diabetes, liver diseases and cancer)

**Supplementary Table 7** Discriminatory performance of clinical models incorporating RAR and MAR for identifying CKD

| **Model** | **AUC (95% CI)** | **ΔAUC** | **DeLong P** |
| --- | --- | --- | --- |
| Clinical model | 0.773 (0.77-0.78) |  |  |
| RAR | 0.781 (0.77-0.79) | 0.008 | < 0.001 |
| MAR | 0.775 (0.77-0.78) | 0.002 | 0.001 |

AUC, area under the receiver operating characteristic curve; CI, confidence interval; ΔAUC, change in AUC compared with the clinical model.

DeLong test was used to compare differences in AUCs between models.

# **Supplementary Table 8.** Hemoglobin and NLR mediating the association between RAR and CKD (n = 27,072)**.**

| **Outcome** | **ACME** | | | **ADE** | | | **Total effect** | | | **Proportion mediated** | | |
| --- | --- | --- | --- | --- | --- | --- | --- | --- | --- | --- | --- | --- |
|  | Estimate | (95% CI) | p | Estimate | (95% CI) | p | Estimate | (95% CI) | p | Estimate | (95% CI) | p |
| **Hemoglobin** | | | | | | | | | | | | |
| Unadjusted | | | | | | | | | | | | |
| Total, N = 27072 | 0.007 | [0.00,0.01] | <0.001 | 0.026 | [0.02,0.03] | <0.001 | 0.034 | [0.03,0.04] | <0.001 | 0.212 | [0.15,0.27] | <0.001 |
| Male, N = 13248 | 0.005 | [0.00,0.01] | <0.001 | 0.008 | [0.00,0.01] | <0.001 | 0.013 | [0.01,0.02] | <0.001 | 0.364 | [0.32,0.40] | <0.001 |
| Female, N = 13824 | 0.006 | [0.00,0.01] | 0.088 | 0.052 | [0.04,0.06] | <0.001 | 0.058 | [0.05,0.07] | <0.001 | 0.102 | [0.01,0.21] | 0.088 |
| Adjusted | | | | | | | | | | | | |
| Total, N = 27072 | 0.018 | [0.01,0.02] | <0.001 | 0.042 | [0.04,0.05] | <0.001 | 0.059 | [0.05,0.00] | <0.001 | 0.298 | [0.24,0.36] | <0.001 |
| Male, N = 13248 | 0.012 | [0.01,0.02] | <0.001 | 0.033 | [0.03,0.04] | <0.001 | 0.045 | [0.04,0.05] | <0.001 | 0.270 | [0.20,0.33] | <0.001 |
| Female, N = 13824 | 0.022 | [0.01,0.03] | <0.001 | 0.049 | [0.04,0.06] | <0.001 | 0.071 | [0.06,0.08] | <0.001 | 0.311 | [0.20,0.44] | <0.001 |
| **NLR** | | | | | | | | | | | | |
| Unadjusted | | | | | | | | | | | | |
| Total, N = 27072 | 0.003 | [0.00,0.01] | <0.001 | 0.030 | [0.03,0.03] | <0.001 | 0.033 | [0.03,0.04] | <0.001 | 0.093 | [0.07,0.11] | <0.001 |
| Male, N = 13248 | 0.002 | [0.00,0.01] | <0.001 | 0.010 | [0.01,0.01] | <0.001 | 0.012 | [0.01,0.02] | <0.001 | 0.175 | [0.15,0.20] | <0.001 |
| Female, N = 13824 | 0.003 | [0.00,0.01] | <0.001 | 0.055 | [0.05,0.06] | <0.001 | 0.058 | [0.05,0.07] | <0.001 | 0.058 | [0.04,0.08] | <0.001 |
| Adjusted | | | | | | | | | | | | |
| Total, N = 27072 | 0.006 | [0.00,0.01] | <0.001 | 0.053 | [0.05,0.06] | <0.001 | 0.060 | [0.05,0.07] | <0.001 | 0.108 | [0.08,0.13] | <0.001 |
| Male, N = 13248 | 0.005 | [0.00,0.01] | <0.001 | 0.040 | [0.03,0.05] | <0.001 | 0.045 | [0.00,0.05] | <0.001 | 0.103 | [0.07,0.13] | <0.001 |
| Female, N = 13824 | 0.007 | [0.00,0.01] | <0.001 | 0.065 | [0.06,0.07] | <0.001 | 0.072 | [0.06,0.08] | <0.001 | 0.097 | [0.07,0.13] | <0.001 |

Abbreviations: BMI, body mass index. CKD, chronic kidney disease. RAR, red blood cell distribution width (RDW) -to- albumin ratio. Albumin is measured in g/dL, NLR was obtained by calculating the ratio of neutrophils count to lymphocyte. Both RAR and NLR were transformed to natural logarithms in the analysis.

a. Crude Model: Unadjusted.

b. Adjusted model: Adjust for age, race/ethnicity, education level, marital status, physical activity, BMI, smoking status, alcohol status and other diseases (hypertension, diabetes, liver diseases, cancer).

## **Supplementary Table 9.** Hemoglobin and NLR mediating the association between MAR and CKD (n = 27,072)**.**.

| **Outcome** | **ACME** | | | **ADE** | | | **Total effect** | | | **Proportion mediated** | | |
| --- | --- | --- | --- | --- | --- | --- | --- | --- | --- | --- | --- | --- |
|  | Estimate | (95% CI) | p | Estimate | (95% CI) | p | Estimate | (95% CI) | p | Estimate | (95% CI) | p |
| Hemoglobin |  |  |  |  |  |  |  |  |  |  |  |  |
| Unadjusted Total N = 27072 | -0.009 | [-0.01,0.00] | <0.001 | 0.897 | [0.87,0.91] | <0.001 | 0.888 | [0.86,0.91] | <0.001 | -0.010 | [0.00,0.01] | <0.001 |
| Adjusted Total N = 27072 | 0.050 | [-0.03,0.00] | <0.001 | 0.578 | [0.44,0.70] | <0.001 | 0.628 | [0.50,0.74] | <0.001 | 0.080 | [0.04,0.12] | <0.001 |
| NLR |  |  |  |  |  |  |  |  |  |  |  |  |
| Unadjusted Total N = 27072 | 0.049 | [0.03,0.04] | <0.001 | 0.840 | [0.80,0.87] | <0.001 | 0.889 | [0.86,0.91] | <0.001 | 0.054 | [0.04,0.08] | <0.001 |
| Adjusted Total N = 27072 | 0.042 | [0.03,0.06] | <0.001 | 0.599 | [0.47,0.71] | <0.001 | 0.641 | [0.51,0.75] | <0.001 | 0.065 | [0.04,0.10] | <0.001 |

Abbreviations: BMI, body mass index. CKD, chronic kidney disease. RAR, red blood cell distribution width (RDW) -to- albumin ratio. Albumin is measured in g/dL, NLR was obtained by calculating the ratio of neutrophils count to lymphocyte. Both RAR and NLR were transformed to natural logarithms in the analysis.

a. Crude Model: Unadjusted.

b. Adjusted model: Adjust for age, race/ethnicity, education level, marital status, physical activity, BMI, smoking status, alcohol status and other diseases (hypertension, diabetes, liver diseases, cance
